# Supplementary material for: The interactome of intact mitochondria by cross-linking mass spectrometry provides evidence for coexisting respiratory supercomplexes
Source: Mol Cell Proteomics. 2017 Dec 8;17(2):216–32. doi: 10.1074/mcp.RA117.000470 (PMC5795388; doi:10.1074/mcp.RA117.000470)
Supplement: Supplemental Data [file supp_RA117.000470_133922_0_supp_23978_fzffwf.zip › spectra_annotation/mito_spectra_annotation/142-1-18-1-14-1.pdf]

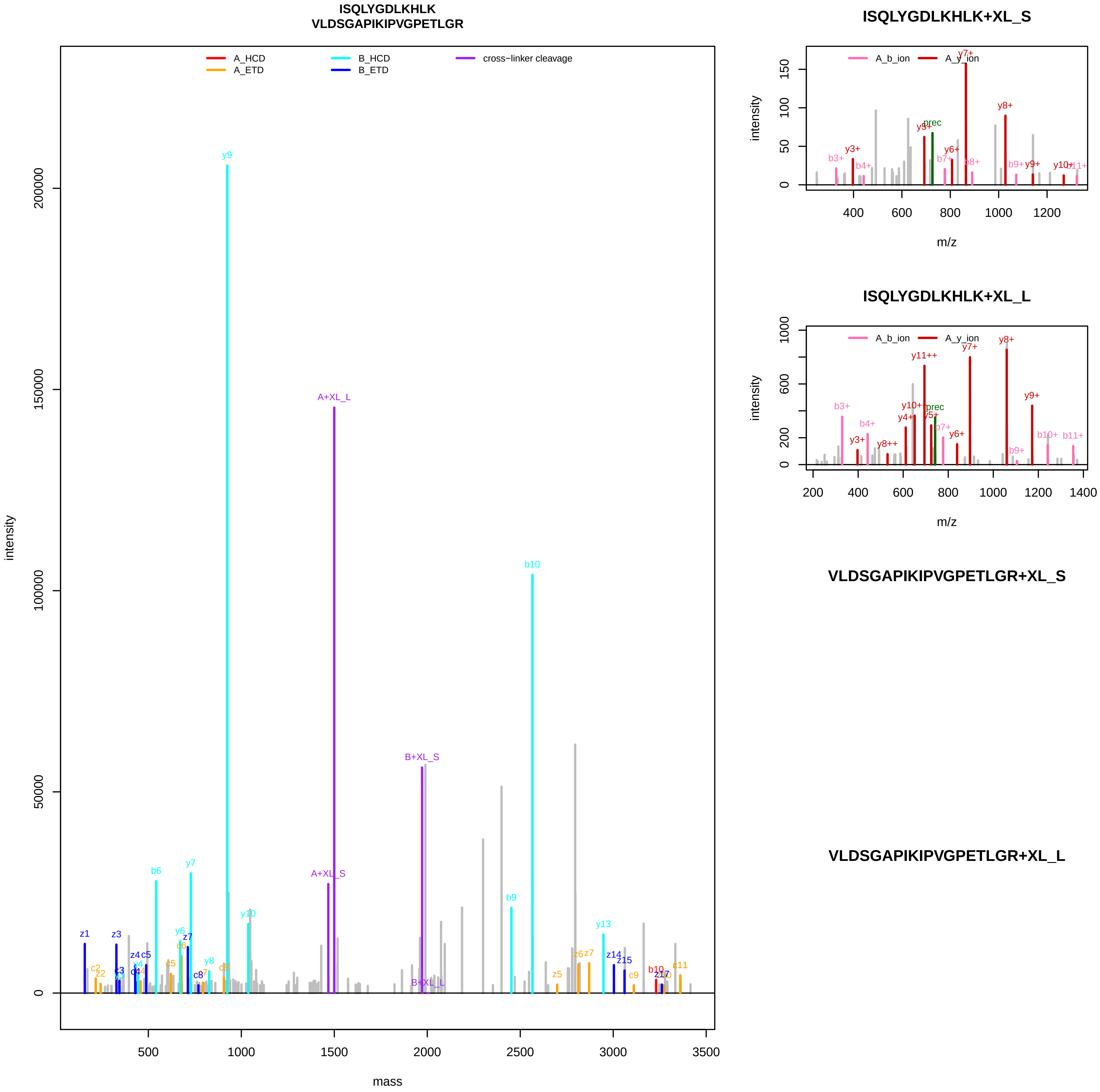
ISQLYGDLKHLK
VLDSGAPIKIPVGPETLGR
A_HCD
A_ETD
B_HCD
B_ETD
cross−linker cleavage
y9
A+XL_L
b10
B+XL_S
A+XL_S
B+XL_L
b6
y7
y10
b9
y13
y6
y8
z1
z3
z4
c5
z7
c8
c2
z2
c3
c4
y4
c6
c5
c8
z5
z6
z7
z14
z15
c9
b10
z17
c11
intensity
mass
0
50000
100000
150000
200000
500
1000
1500
2000
2500
3000
3500
ISQLYGDLKHLK+XL_S
A_b_ion
A_y_ion
prec
y7+
y8+
y5+
y3+
y6+
b3+
b4+
b7+
b8+
b9+
y9+
y10+
b11+
intensity
m/z
0
50
100
150
400
600
800
1000
1200
ISQLYGDLKHLK+XL_L
A_b_ion
A_y_ion
y8+
y7+
y11++
y10+
prec
y5+
y4+
b3+
b4+
y3+
y8++
b7+
y6+
y9+
b9+
b10+
b11+
intensity
m/z
0
200
600
1000
200
400
600
800
1000
1200
1400
VLDSGAPIKIPVGPETLGR+XL_S
VLDSGAPIKIPVGPETLGR+XL_L
